# Exploring the Etiological Pathways of Problematic Pornography Use in NoFap/PornFree Rebooting Communities: A Critical Narrative Analysis of Internet Forum Data

**DOI:** 10.1007/s10508-021-01930-z

**Published:** 2021-06-18

**Authors:** Dimitra Chasioti, James Binnie

**Affiliations:** Division of Psychology, School of Applied Sciences, London South Bank University, 103 Borough Road, London, SE1 0AA UK

**Keywords:** Problematic pornography use, Critical narrative analysis, Online self-help communities, Rebooting communities, NoFap/PornFree

## Abstract

Problematic pornography use (PPU) has been extensively studied in terms of its negative implications for various life domains. The empirical literature reveals measured outcomes of interpersonal and intrapersonal dysfunction in participants’ everyday living, supporting its classification as a disorder. The increasing number of complaints around PPU opens the door to the creation of online self-help rebooting communities. This qualitative study aimed to provide a better understanding of this behavior by investigating potential etiological pathways contributing to the onset of PPU, as they were expressed by members of the online NoFap/PornFree self-help communities with self-perceived PPU. The critical narrative analysis reveals a complex web of mutually informing causal connections. The dialectical relationship between situational resources, material conditions, and an embodied spectator gives rise to an online persona with motivations of self-exploration, experimentation, and socializing. A sense of vulnerability rendered the use of pornography as a means of escape and validation. Furthermore, commitment to abstinence, framed by the notions of recovery and relapse, was found to be a major factor for maintaining distress. The study highlighted the need for a thorough understanding of the etiological pathways of PPU for a more effective and targeted intervention. Moving beyond biomedical conceptualizations suggests an intervention whereby PPU is placed in a context of a crisis of meaning.

## Introduction

### Pornography in Context

#### Defining Pornography

The definition of what actually constitutes pornography is a necessary step preceding any discussion related to pornography. Some of the main contradictory viewpoints have drawn upon liberal and radical feminist epistemologies, or have moral conservative roots (McNair, 1996). These approaches created dichotomous interpretations (aesthetic-offensive, acceptable-obscene, normal-commercial), influential to sexual policy (Allen, 2001).

Defining pornography can also be conceptualized more individually, by focusing on the kind of pornographic material designed to stimulate the consumer (Kor et al., 2014). This “stimulus-effect” approach is often used and leads to pornography being defined as any sexually explicit material (SEM) aimed towards sexual arousal (Kohut et al., 2020; Marshall & Miller, 2019). This is the definition to be adopted throughout this study.

#### Internet Pornography

28,000 users are seeking SEM on the internet every second (Hessen & Pedersen, 2017). In terms of the way that pornography is used, the majority of studies clearly link male usage with masturbation practices whereas female usage is often presented as a shared leisure activity with their sexual partners (Sørensen & Kjørholt, 2007). However, more recent research has revealed a higher probability of individual private use for both men and women (Kohut, Fisher, & Campbell, 2017).

Internet Pornography (IP) has been theoretically characterized by its accessibility, affordability and anonymity (Cooper, 1998); King (1999) later added the factor acceptability and Hardy (2008) interactivity. Developments in technology and culture have resulted in the modification of the pornography industry and user’s online identity; from consumer to vendor (Attwood, 2002). These innovative features of the internet have contributed to the democratization of pornography, shifting pornography from an “obscene” to an “on-scene” interaction (Escoffier, 2007) and the pornography user from the “otherness” to “one of us” (Attwood, 2006).

#### Internet Pornography and Related Effects

Within discussions around the expansion of the qualities and capacities of IP, a discourse of dangerousness arising from its consumption emerged. By the end of 1990s, alongside the media, there has been a proliferation of academic and clinical literature investigating the potential effects of IP on the user’s mental wellbeing (Delmonico, 1997).

The use of IP has been associated with a negative impact on the user’s interpersonal relationships, however this very much depends on the meaning attached to pornography use (Poulsen, Busby, & Galovan, 2013). Studies have suggested that when women discover their male partner’s IP use there are feelings of betrayal, exclusion, and isolation (Bergner & Bridges, 2002); and insecurity, lack of self-esteem, and trust, and a decrease in the quality of the relationship (Bridges & Morokoff, 2011). Describing this “third-person” effect, Lee and Tamborini (2005) suggested that the negative outcomes of IP within relationships can be experienced, mostly, by people close to online users rather than by the users themselves. Among other identified problems of an interpersonal nature associated with IP use are references to vocational problems (Wright & Randall, 2012; Young, 2007), family dysfunction (Manning, 2006), and interpersonal isolation (Yoder, Virder, & Amin, 2005). Despite these issues it has been suggested that standard hardcore pornography is not directly “harmful” to the user, however on balance there are few benefits associated with it either (Binnie & Reavey, 2020).

### Problematic Pornography Use

#### Classification

Patterns of behavioral, intrapersonal, and interpersonal effects within the individuals’ narratives and also studies reporting individuals’ unsuccessful efforts to reduce their use of IP, (Young, 2007,2008) have boosted assertions of the notion of Problematic Pornography Use (PPU), fueling a debate around definitions and models in an attempt to classify this behavior.

Several types of nomenclature have been proposed in order to define this phenomenon. Compulsivity (Cooper, Scherer, Boies, & Gordon, 1999), impulsivity (Shapira et al., 2003), and hypersexual disorder (Kaplan & Krueger, 2010) are suggested analytical frameworks. PPU is informally regarded by many as belonging to the spectrum of behavioral addictions (Pinna et al., 2015); currently, in the International Classification of Diseases (ICD-11), PPU can be included either under the diagnostic spectrum of compulsive sexual behavior disorder or under the umbrella of “other specified disorder due to addictive behaviors” (Grubbs et al., 2020).

Subsequently, the clinically observed behaviors of repetitive and persistent urges and the overwhelming emotions linked with abstinence, craving, tolerance and withdrawal, urged some scholars to draw parallels between IPU and substance-related addictions. This phenomenology of characteristics spurred the creation of the biomedical model of pornography addiction (Duffy, Dawson, & Das Nair, 2016). However, these similarities have also been the main line of criticism against the use of this disease model of PPU with Ley, Prause, and Finn (2014) arguing that the lack of validity and reliability of the addiction model lies in its weakness to make distinctions among clinical cases.

Other types of criticisms refer to the subjectivity of using specific variables. For instance, it has been argued that the notion of “frequency” is used arbitrarily. It should also be noted that in some cross-sectional studies variables such as non-heterosexual orientation, morality, and/or high sexual drive seem to be disregarded (Ley et al., 2014), raising assumptions for moralistic judgments, suppression of sexual minorities and oppression of free speech (Clarkson & Kopaczewski, 2013).

Cavaglion’s (2009) narrative analysis of 2000 posts of men struggling with their IP use identified multiple forms of distress. The users’ experiences included significantly reduced self-esteem, overwhelming emotions arising from their difficulties in managing their personal lives, and sexual problems. Additionally, in their exploratory investigation regarding IP’s impact on individual’s wellbeing, Philaretou, Mahfouz, and Allen (2005) found that the majority of interviewees stated feelings of psychological distress; considerable guilt, anxiety and depression were particularly mentioned which in turn led to great difficulty and/or inability to experience intimacy with their real-life partners. However, as Grubbs, Stauner, Exline, Pargamen, and Lindberg (2015) highlight, research exploring associations between IPU and emotional distress could not be deemed as representative. Mediating factors, identified by other studies, such as experiential avoidance (Levin, Lillis, & Hayes, 2012), relational conflict (Gwinn, Lambert, Finchman, & Maner, 2013), and moral incongruence (Grubbs & Perry, 2019; Perry & Whitehead, 2018) have not been taken into account while the potential positive effects of IP remain under investigated.

Employing similar methods but being epistemologically opposed to Cavaglion’s (2009) conceptualizations of pathology, Taylor and Jackson (2018), revealed that the assumptions regarding PPU were directly associated with the socially constructed ideals of masculinity. Particular mention should be made to Grubbs, Exline, Pergament, Volk, and Lindberg (2017) who added the factor of the self-perceived addiction within the debate over the effects of pornography. They supported that it is religiousness and spiritual dissonance that may lead users to experience themselves as pornography addicts rather than pornography use per se.

Overall, there is a debate over the labelling of “excessive” IPU based on disagreement over objective behavior, subjective experiences, and the way in which these experiences are framed analytically by research. For the purposes of this research, the notion of PPU will be defined as any use of pornography that leads to significant negative interpersonal, intrapersonal and extrapersonal consequences of the use (Sniewski, Favrid, & Carter, 2018). However, despite the over-documentation of PPU’s negative effects, the question arises as to what distinguishes one’s normative involvement in IPU from potential pathological aspects of this behavior. Therefore, the examination of the underlying psychological causes for the onset of PPU is considered of utmost importance.

#### Problematic Pornography Use and Associated Etiological Pathways

Potential etiological pathways regarding the onset of PPU could be attributed to interpersonal issues. Ybarra and Michell (2005) report physical and sexual victimization as well as poor emotional bonding with childhood caregivers enhancing assumptions for a possible correlation between insecure attachment styles and PPU. By placing PPU into the wider context of internet addiction, it can be suggested that insecure attachment, framed with patterns of a distributed parental environment (Şenormancı, Şenormancı, Güçlü, & Konkan, 2014) and domestic violence (Park, Kim, & Cho, 2008), can be positively linked with participants’ vulnerability to internet addiction. Moreover, Thomas (2016) approached cybersex as an issue stemming from an insecure environment; in particular, drawing on humanistic underpinnings, she indicated cybersex as a need for affirmation and connection with others in a non-threatening space. Subsequently, IPU was a respond to individual’s feelings of loneliness and social impairment, operating as a mirror of an idealized online self.

Considering intrapersonal factors as potential contributors to the onset of PPU, Cooper, Griffin-Shelley, Delmonico, and Mathy (2001) suggest that internet functioned as a means for users to explore pre-held sexual fantasies or as way to alleviate their stress; the majority of participants who mentioned previous experience of distress and anxiety used IP frequently as a form of relief. Several studies (e.g., Cooper, 1998; Young, 2008) have attempted to demonstrate a statistically significant link between overwhelming emotions and PPU, framing the use of IP as a coping strategy.

Besides this self-medication hypothesis (Khantzian, 1985), Grubbs, Perry, Wilt, and Reid’s (2019) theoretical model of PPU puts forward an etiological pathway whereby moral disapproval of pornography lead to moral incongruence (the feeling that one’s values have been violated). In that way, they suggested the perceptions attached to one’s IPU as a valuable factor to examine distress around PPU, a hypothesis that was confirmed later both in Perry’s (2019) and Sumerau, Cragun, and Barbee’s (2018) qualitative research. Even if moral incongruence was a radically introduced term in exploring self-perception of PPU, Burke and Haltom’s (2020) qualitative project broadens the discussion in the field of religious research and PPU by highlighting the importance of the power of gender theory. The analysis of 35 interviews with conservative religious participants in porn-addiction recovery settings revealed that participants drew on essentialist discourses, informed by religious schemas, in order to explain, scientifically, their addiction to pornography. Participants’ narratives of being biologically preconditioned by God to IP use suggested hybrid masculinity as a perceived causal pathway to PPU.

This determinist rationale in relation to PPU meaning-making, was introduced by Taylor and Jackson’s (2018) discourse analysis on online members’ posts of the self-help community, NoFap. Masculinity as both “innate” and performance was revealed as the dominant discourse on investigating the masculine spectrum of subjectivities that are constructed by the context of Nofap. Following the same epistemological background, yet shifting the research focus from porn to a masturbation perspective, Hartmann (2021) tried to investigate “how one becomes someone”(p. 3) by analysing NoFap videos on Youtube. The critical discourse analysis indicates that masturbation-abstinence talk promotes specific forms of self-relation connected with manospherian rationales, promoting a sexual work ethic. Considering that the masturbating male acquires effeminate characteristics—as they lost their natural agency of heterosexual intercourse—the abstinent male should self-govern one’s body in order to reconcile them with their natural sexual existence.

Drawing on gender studies and recent sociological research, these qualitative projects updated the framework around the PPU study while emphasizing the great influence of evolutionary psychology in shaping personal narratives. Even if the research focus is not on the potential causal pathways of PPU, masculine heterosexual performance is indicated, indirectly, as a potential hypothesis involved in the onset of PPU.

Finally, Cavaglion’s (2009) qualitative research revealed, indirectly, references of predisposing factors in the onset of PPU. Through narrative analysis, voice was given to men’s lived experiences. All of the narrators, divided into two groups according to their emotional and demographic characteristics, expressed overwhelming emotions about their use and its devastating effects in their lives. The first group explicitly mentioned patterns of isolation and solitude since childhood, while the second one referred to a specific stressful event (divorce, breakups, unemployment) followed by the consumption of IP. This latter group seems to draw upon the disease model, whereby “external” factors are presented as environmental triggers, contributing to individual’s onset of a pathological behavior regarding their use of IP.

### The Current Study

Attempts have been made to understand the potential causes of PPU by making indirect links with developmental, interpersonal and psychological aspects. However, the majority of these studies do not focus on PPU per se as they draw heavily on conceptualizations of behavioral (internet or sex) addictions (e.g., the I-PACE and the Sexhavior Cycle model; for reviews, see Brand, Young, Laier, Wölfling, & Potenza, 2016; Walton, Cantor, Bhullar, & Lykins, 2017, respectively). PPU can be considered a truly complex phenomenon, highlighting the necessity to be investigated beyond the narrow limits of quantitative methodologies. It can be argued that the lived experience of problematic pornography users has been largely ignored when compared to the volume of studies concerning classification and measurement. Moreover, the existing qualitative research is more concerned in exploring PPU from a masturbation and abstinence perspective in an attempt to gain a better understanding of the NoFap culture as well. In particular, the investigation of the ways in which IP and self-help online community users reflect on their perceived onset of PPU is lacking within the academic literature, a dimension which will be the focus of this study. Therefore, this investigation proposes the following research question:

How do members of Nofap/PornFree online support communities express and inform their narratives regarding the etiological factors of their self-perceived Problematic Pornography Use?

## Method

In this qualitative study an online ethnographical approach for data collection was used. Specifically, the data set was written material gathered from online asynchronous discussion forums: responses to posts were exchanged over an extended period of time. Online discussion groups allowed the researcher to adopt a passive approach, as they did not participate actively in the discussions. This feature can be linked to the naturalistic data collection model (Eysenbach & Till, 2001).

### Participants

The website Reddit, a social news and media aggregation website, served as the gateway for the study. Registered users interact by commenting, rating, posting and discussing on various topics of interest. In turn, these exchanges create several types of communities with their own interaction dynamics, rules, and vocabulary (Taylor & Jackson, 2018), called “sub-reddits” hosted within Reddit.

This work was conducted using posts (threads) from the subreddits NoFap (see r/NoFap) and Pornfree (see r/pornfree). These forums are available 24 h a day, publicly accessible for reading, while posting is allowed only via registration. Anonymity is preserved by using usernames. These sites seem to share the same philosophy; IPU is considered unhealthy and has severe effects in relational and personal domains. The notion of pornography addiction is clearly manifested in both of these self-help community forums; with recovery as their stated aim, regarded as attainable mostly through abstinence from pornography and/or masturbation. However, it needs to be mentioned that in contrast to the strict guidelines of r/Nofap regarding abstinence from pornography, masturbation, and orgasm (PMO), the subreddit r/pornfree seems to support the abstinence from pornography-induced masturbation without criticizing necessarily the masturbation itself. Given the person-centered approach interwoven in these forums, mutual support is achieved through encouraging answers, motivational tracts, information sharing and consultation for alternative approaches. Based on asynchronous posting in conversation threads, these forums provide vast numbers of shared stories of IPU’s effects on their daily lives as well as around the potential roots of their situation.

For this study, participants were 40 registered online forum members (30 from r/Nofap and 10 from r/pornfree respectively) who described themselves as problematic pornography users or pornography addicts. Throughout the manuscript, participants share problems and report difficulties related to both perceived addiction to pornography and perceived pornography induced sexual difficulties (PIED). Out of 40 participants, 26 mention erectile dysfunction (ED) and/or porn-induced ED as their main topic related to self-perceived PPU while 14 of them make complaints about porn addiction without mentioning any sexual difficulties. Despite the fact that sociodemographic details were not easily accessible due to the anonymity provided on the internet, information on age and gender was deduced implicitly from the content of posts (e.g., “I am a man, 21 years old”). Thus, in this study the majority of participants were males between 21 and 32 years old, heterosexual (e.g., “I broke up with my girlfriend 2 years ago”) with good intellectuality (the majority of them being university students and/or employed individuals). Some of them could be considered as actively engaged in the forums while others were more occasional users. Moreover, a degree of heterogeneity about their cultural background could be assumed based on expressions like “English is not my first language.”

The sample investigated can be seen as representative to the forums as a whole. NoFap’s (2014) survey revealed that heterosexual (94%) males (99%) between the ages of 17–28 (78%) are the predominant population on their sites. To our knowledge, there is no similar research that shares the demographics of r/pornfree’s online members so far.

### Ethical Considerations

In spite of the fact that online forum communities are widely recognized as a valid method for research (Montgomery & Gottlieb-Robles, 2006), there is an ongoing debate over their public or private nature (Jowett, 2015) followed by considerations concerning the traceability of the data (Rodham & Gavin, 2006). Since r/Nofap and r/pornfree are freely accessible as participants’ threads are classified in the public domain (Smithson, 2015), the need for consent was considered desirable but not mandatory. Thus, to further enhance transparency, the website developers were approached and permission for data collection was given by the Reddit support team.

Additionally, in order to protect online user privacy, the existing usernames and pseudonyms were replaced and the verbatim quotes presented within the study have been paraphrased to the extent that they cannot be traced back to the original source (Hewson & Buchanan, 2013).

### Procedure

The process of data collection took place following the typical linear process of a passive Internet-mediated ethnographical approach: observing, downloading, and analyzing posts on web-forum discussions (Morison, Gibson, Wigginton, & Crabb, 2015). Data were collected using a password protected computer. A 4-month period was spent reviewing the online forums and reading multiple posts in order to become familiar with the structure, language and the conversational styles of the forums. The subreddits’ search/filter function was used to explore threads of interest using the key words “causes,” “my story,” “history,” “roots,” “contributing” which afterwards were used together (e.g., “contributing and story”). These words were then combined with specific developmental stages: “causes and childhood” and “causes and adolescence.” As this method resulted in a multitude of threads, additional limitations for data management were adopted. In particular, the time-posting period was set within the last 5 years (2013–2018) while posts under the length of 200 words were excluded. The emphasis was placed on narrative stories with detailed references to the beginning of engagement with SEM, descriptions of events and/or factors that contributed to the shift from pornography use to experiences of PPU. Narratives that revealed personal accounts of psychological processes, interpersonal and intimate relationships, and/or bodily experiences related to the onset of PPU were included. In contrast, posts regarding experiences, goals and progress of abstinence, and narration concerning pornography addiction effects were excluded along with advice for new methods of controlling sexual urges.

This process led to the gathering of a 114 page data set consisting of 53 narrative stories/participants. In a finalizing step, the researcher went back to each narrator’s posting history of threads. Thus, based on the same inclusion criteria, relevant threads were included to create a coherent story for each participant. Participants that did not have a threads’ history were excluded. Within the first draft of data set, 48 online members were involved, while the number of posts to be analyzed amounted to 121. After the initial coding process the focus was centralized on screening across various domains of experience, looking for commonalities and differences among the main themes. Participants’ stories that were not representative of the prevailing narratives were excluded. The final data set that contributed to the results consisted of 40 participants and 100 posts.

### Data Analysis

This study employed a qualitative methodology to give voice to individuals with PPU in order to explore the multidimensional nature of their experiences, feelings and individual perspectives. Drawing upon the epistemological underpinnings of social constructionism, the emphasis is placed on how individuals’ representations and knowledge are constructed socially, historically, culturally and discursively (Burr, 1995; Gergen, 1999). Within this context, critical narrative analysis (CNA; Emerson & Frosh, 2004) was utilized for the analysis of this study. CNA explores storytelling both in their descriptive and performative function, that is, “why the story was told that way” (Riessman, 2001, p. 74). Therefore, the emphasis is placed upon the narrators’ voices (experiences, feelings, and behavior) and discourse choices (verbal actions, influenced by wider social-cultural practices that form and inform the experiences described) (Chase, 2005).

In contrast to other research methods, there are no agreed guidelines for narrative analyses (Andrews, Squire & Tamboukou, 2013). For analytical purposes and to avoid possible fallbacks to “retelling the story,” the data analysis was conducted by using Murray's (2000) four analytical levels of narrative: personal, interpersonal, positional and ideological. Emphasis was initially given on the understanding of participants’ narratives of their experiences and descriptions of meaning-making in their effort to make sense of their PPU. The interpersonal level essentially recognized the contribution of the wider audience to the process of meaning- making. Based on that, the forums’ values and theoretical orientations were taken into account in relation to how narrators inform their sense-making process. For practical purpose, the latter two levels are incorporated in one “public narrative” (Murray, 2000). These aspects allow for a deeper understanding of broader social and cultural contexts underlying moral functions and shared-representations of the online users’ repertoires (Stephens & Breheny, 2013).

Murray’s integrated approach was implemented alongside a typical coding process. Initially, an examination of the data was conducted repeatedly leading to provisional codes. Alongside this stage, an attempt of summarizing the crucial elements of each narrator’s story took place. Proceeding to a further stage of data engagement, significant patterns and concepts were identified while personal reflections on them (memos) were created. These patterns were grouped, in turn, into themes which were finalized after repeated modifications. Furthermore, the identified stories and canonical narratives were compared across individuals. Differentiations and commonalities were framed using Murray’s narrative lenses. Across the analytical procedure emphasis was placed on the co-construction of the identity and the experience. This level of synthesis led to the creation of a coherent whole. Before the final stage of writing up, a checking process of the internal homogeneity and the external heterogeneity (Patton, 1990) of the themes took place to evaluate the validity of the final data. Specifically, it was assessed if all the quotes of each narrator convey a coherent pattern and whether the themes reflect the meaning included in the whole data set.

## Results

**Fig. 1:**
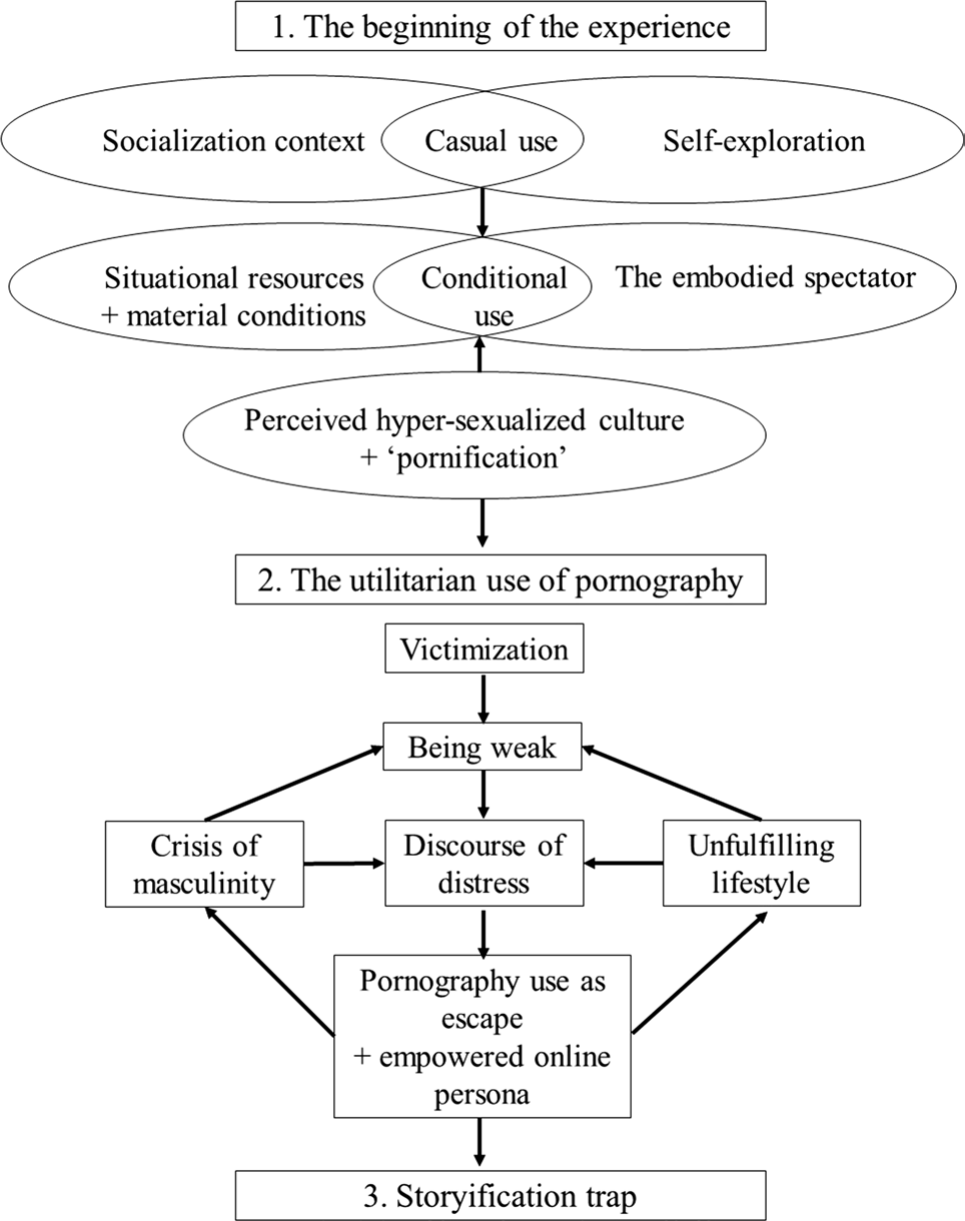
Summary of the proposed etiological connections of self-perceived PP

In this section, the prevailing narratives of participants’ are theoretically presented as a three part chronicle. These categories are distinct in their nature but overlap, to a great extent, in terms of their mutually informing causal interactions. Across the chronicle, the three analytical levels are intertwined and work together to provide a coherent and prevailing story. A summary of the potential etiological factors, as constructed from the analytical process, is illustrated in Fig. 1.

### The Beginning of the Experience

**[Extract 1]**: I have been masturbating since I was 11–12. Everything started when a friend gave me some CDs with hardcore content… Since then I have been masturbating every day… When I was 15 I got a limitless internet connection, and that's when all began to turn serious (participant No. 1, lines: 8–11).

**[Extract 2]**: Alone now, out in the world, with my own paycheck to satisfy myself with whatever I wanted. Not concerned about my father catching me, not worried about friends finding my secret stash at school, I could consume as much as I wanted for as long as I wanted (No. 33, 3091–3095).

The above extracts exemplify the typical starting point of participants’ personal story regarding IP use. The participants’ accounts provide a chronologically structured ontological narrative where specific space and place relationalities introduce their engagement with IP use as a multifaceted, context-created temporal experience.

As it is indicated, the onset of their use began in the early or middle adolescence, an event which is representative across the data set. Within this context, pornography was introduced, in part, by peers in a socialization context. Extract’s 1 positioning regarding the initial phase of IP use illustrates that pornography use is a matter of privacy, open for sharing with a social group. This public narrative implies the participants’ casual use, motivated and framed by the longing for bodily self-exploration. However, participants’ discourses around availability of resources, experimentation and privacy frame the shift from casual pornography engagement to more conditional use. Specifically, the availability of a private space, internet access, and a limited family gaze enhance IP engagement to a great extent. For instance, narrators clearly point out that acquiring a feeling of personal freedom without external limitations was a pivotal moment for its use in a regular basis (Extract 2).

Thus, situational resources (private space and free expression) and material conditions (computer existence and internet access), usually associated with and enhanced by a discourse of virginity, is a crucial factor in initiating engagement with pornography. This context-dependent experience seems to facilitate the creation of personal times and spaces of self-enactment. Within this process of sexual interaction between the body and the screen, where the absence of the “third-other” is necessary, the embodied spectator emerges, deriving pleasure from watching and sexualizing the content on their display. These dimensions, together, are involved in shaping and establishing the identity of the regular pornography user. The image of a person self-restrained to their private space, in the presence of a computer, masturbating with several tabs running simultaneously, is commonly described by participants.

In this particular phase, participants justify their positioning with pornography turning into a matter of “consumption” by referring to culturally established narratives of the ubiquity of pornography within a perceived hyper-sexualized culture [e.g., “porn is everywhere” (No. 7, 668), “scantily-clad ladies are everywhere” (No. 10, 1006)]. At the same time, the decisive role of this social phenomenon in the onset of PPU is revealed:

**[Extract 3]**: Every time I see something somewhat sexual, I turn into an autopilot mode and spend lot of time on it. I really have no idea how to stop, even though I'm aware of what's happening (No. 8, 758–760).

**[Extract 4]**: In short, when you spot a very hot image, be it on a screen or in real life, it says: “I am what you want.” And your normal answer is: “Yes! Yes! I want you! I want more of this!” At that very moment you’re screwed up (…) you’re getting on a train moving toward one more failure (No. 22, 1735–1739).

This process of “pornification”—where pornography is gradually incorporated in everyday life by becoming normalized in public life (Mulholland, 2011)—is interpreted by narrators in terms of a “stimulus–response” interaction that has been familiarized and internalized afterwards. This behavioral discourse, combined with the patterns of “sickness” [e.g., “It's the current society on the whole that is sick” (No. 22, 1797–1798)] and “demonization” for describing society [e.g., “You see, somewhere along the way I decided pornography was the devil” (No. 23, 1835)], essentially reflects the dynamics of this phenomenon as an integral part of everyday life. At the same time, these discursive choices are utilized by online users in order to construct themselves as passive receivers on the basis of “I am what you want”; adopting an external locus of control for their pornography use. In that way, a pleasurable “auto-sexuality” is being formed, based mostly on male hedonism and female exhibitionism (Attwood, 2010).

Overall, the dialectical relationship among these factors at the beginning of the experience suggest the core context of emerging stories, practices and performances onto which participants’ rationale of self-perceptions of PPU is being built.

### The Utilitarian Use of Pornography

**[Extract 5]:** We've all been raised in an oversexualized culture and the weaker of us have fallen into the trap of today’s possibilities and been screwed up by excessive fapping^1^ or seeking unnatural amount of arousal (No. 22, 1783–1785).

This part of the chronicle includes the narrations of participants regarding the meaning they attach to IPU as well as the way its use frames their lived experience. As stated above, the encouragement for pornography consumption from wider cultural and social structures seems to be recognized by online users. However, the aforementioned quote reveals a causal relationship between the pattern of “being weak” and an individuals’ PPU. In short, what is supported here is that in this pornified culture only the “weak” individuals could be affected to such an extent that their use could be considered as problematic.

Therefore, this section constitutes an investigation of the etiological connections that informs this core causal link, by examining the psychological states, events and processes that construct the identity of “being weak” as they emerged from narrators’ stories.

**[Extract 6]:** We need to be sure we can trust them not to pull any shit. The people on the screen and in my head on the other hand are under my control. I don't have to be intimidated by them. Instead I get to "intimate" them. Similar word, different meaning. My brother and cousin broke me long ago by physically dominating me, keeping me in a prolonged state of fight-or-flight, never able to let my guard down (…) and then others from school latched onto that pattern, until I was totally consumed by self-loathing. I decided to rely on fantasy, porn, fapping, and weed instead of real relationships from now on (No. 24, 1881–1888, 1988–1991).

**[Extract 7]:** I don’t know how all began. I am pretty sure it actually started when girls started to call me ugly. Afterwards I somehow turned to porn (No. 3, 169–170).

A prevalent pattern across individuals’ sense-making process is a history of victimization (bullying), especially within their environment of interaction. This victimization takes the form of physical abuse, taunting behavior, and social isolation by peers. In their narrations, many participants (specifically 16 out of 40) reported complaints about intimidation and fears for their safety when exposed around others. These feelings of insecurity are also perpetuated by a sense of lack of control over the situation, which in turn enhances the narrators’ sense of dangerousness (e.g., “keeping me in a prolonged state of fight-or-flight”- Extract 6). This prolonged psychological state of vulnerability might consequently lead to problems regarding trust. Thus, it could be asserted that this core sense of security crisis (sense of meaning, predictability, safety, and control) constitutes one of the dimensions of “being weak.”

The participants’ lived experiences of intense and prolonged states of extreme shame and worthlessness lead to an overall desire to hide, suggesting loneliness as a conscious choice to avoid the painful gaze of peers. Here, the notion of loneliness is conceptualized both as self-isolation and lack of emotional intimacy with others within a context of loss of complete control over the situation. Interlinked with these experiences were participants’ accounts of feelings of shame and self-hate, usually accompanied with references of substance misuse (e.g., “This situation is unbearable and people develop all sorts of defences against it. One common defence is to become the failure the person feels to be: drops out of school/worklife, abuses alcohol/drugs” [No. 25, 2104–2106]).

These psychologically discursive repertoires (e.g., “I am totally consumed by self-loathing,” “develop all sorts of defences”) construct a discourse of distress that needs intervention and a temporary solution—an escape from the perceived “problem.” Pornography consumption is regarded as a comfort zone and presented as a non-intimidating practice. On this basis, it can be argued that under these circumstances, pornography usage can be conceived as a psychological form of dissociation, acting as a defence mechanism against the prolonged violation experienced in adolescence.

**[Extract 8]**: Probably the problem stems from my father being emotionally absent from my life for the biggest part of my adolescence… If it is more an outcome of my childhood and the lack of acceptance from my alcoholic father, what can I do with that insight? (No. 29, 2779–2780, 2868–2869).

**[Extract 9]:** The cause is lined to mother and/or father since you were born, who either didn't accept you as you are, always disapproving, controlling the mind forms natural defense/escape pathways to block the pain… this is how addictions form (No. 18, 1528–1532).

Consistent with the use of pornography use as a means of escape are narratives derived from the interpersonal environment of the family. Specifically, mentions of a physically and emotionally distant father, a disturbing environment and/or controlling parents, structure a wider pattern of a non-supportive familial environment. As it is illustrated in narrators’ confessions, these patterns have significantly influenced their perception about their self and self-confidence. In that way, the causal interactions involved in the psychological state of “being weak” are enriched with a new dimension. Feelings of being humiliated, betrayed, deceived and abandoned are the prevalent intrapsychic factors that add to the etiological spectrum of pornography use as a means of escape.

**[Extract 10]:** This is how my psychology works. I feel like a failure in social relationships, then I feel like a smashing success in sexual relationships through the medium of porn and frequent, lengthy masturbation (No. 25, 1958–1960).

**[Extract 11]:** I think porn looks to me like a strong representation of myself. That is, it usually makes perfect sense to me that I would be a kind of sex-god. I use virtual reality as a method of seeing what to me seems like true reality (no 24, 1947–1949).

As it can be assumed from these narratives that the feature of “de-individualization”—“a psychological state in which inner restraints are lost when individuals are not seen or paid attention to as individuals” (Chiou, 2006, p. 548)—offered in pornography consumption, is predictive of the participants’ desire towards fantasizing. Participants, essentially, experience in their imagination, situations that are strongly related to their relational background. These narratives underscore a need to feel secure, to gain a sense of “being in a position of power” (e.g., “sex god”), and succeed in a relational domain. In other words, participants have the opportunity to escape from a painful reality and resort to an artificial imaginary world, where its feasibility enhances their wishes for transformation into an empowered online persona. This narrative illustrates that participants are positioned as both the subject and the object of the sexual intercourse by displaying themselves on the screen and envisioning the role of the actor. Therefore, frequent pornography engagement within this context serves a multitude of purpose; a means of escape from a painful reality, and a way to enhance feelings of self-worth through fantasy.

**[Extract 12]:** So actually a couple of months ago I had a chance to have sex with a girl that I liked for some time, and she was fairly good looking. It was 5 in the morning and I was quite drunk, and I have been able to get a half erection after she pleasured me orally. I blamed it on alcohol and tiredness. I felt shitty and embarrassed. She was quite disappointed as well as she really wanted me to (expletive) her (…). It was 80% at first, but as she continued my soldier started to shrink, and finally ended totally lifeless (No. 1, 23–24, 40–43)*.*

**[Extract 13]:** I just broke up with a 25 year old girl who was more experienced than me. She was very attractive and wanted me BAD. I started to notice that I wasn't able to perform regularly (even though I found her attractive) and it got to the point where it was pissing her off and led her to cheat me. I felt depressed and less of a man because I couldn’t satisfy her hunger for me (No. 2, 146–150).

A dominant narrative that has been observed across participants’ threads is a series of disclosures around the significance of gender performativity highlighting the importance of masculinity reassurance through sexual intercourse. Sexual performance is virtually interconnected with scenarios of interpersonal sexual negotiations and demonstration of masculinity via a successful erection. Through this performative narrative, participants introduce the significance of corporeality in negotiating the longing for gender comfort and consciousness. As a consequence, online users’ narratives of distress stem greatly from their inability to demonstrate sexual prowess, particularly to a desirable and “hungry” woman. This social construct of women as having an unlimited sexual appetite seem to co-construct men as super-powered, always ready to perform successfully. Therefore, participants’ narratives regarding sexual arousal and ability to perform, as well as the meanings attached to them, are structured around prescribed expectations. Thus, these two bodily practices (optimal functioning and partner’s sexual satisfaction), combined with the pattern of sexual inexperience, engender a feeling of failure.

Moreover, the self-perception of sexual inadequacy seems to result in ambivalence around their virility which in turn leads to overwhelming emotions such us deep shame and embarrassment. According to Thomas (1999, p. 131), “in the sexism of heterosexuality the self-identity of men is inextricably tied to their penises.” Thus, its successful function during sexual intercourse is an indispensable manifestation of true manliness. A “real man” is validated through the incorporation of the sexual drive into a bodily practice. Consequently, erectile dysfunction (ED) is followed by an ongoing self-doubt, perceived self-worthlessness, and crisis from lack of control, while being rejected sexually adds to feelings of gender humiliation.

**[Extract 14]**: That was a really difficult time for me. I became very depressed for a long period and I consumed more and more porn, in order to prove to myself that I still can have an erection during fapping (No. 4, 227–229).

**[Extract 15]**: I started watching more porn, as porn is the only thing that helped me get an erection and I was thinking I will be more in-control with her next time. All went in reverse of course (No. 6, 401–403).

In the aftermath of this crisis of masculinity, narrators are turning to pornography consumption in a desperate attempt to confirm their physical adequacy. Since its quasi-realness and enhanced interaction facilitates the self-reflection of the user on the screen, the virtualized “authenticity” of documented successful arousals and orgasms aid the participants to feel better about themselves. In this almost personal relationship with the image, participants’ need to verify that they are “real men” is being artificially met, leading to an increased consumption of pornography. However, this etiological relation between a fragile identity and pornography consumption underscores a feedback loop; pornography’s illusionary ailment of sexual difficulties drives individuals into compulsive use.

**[Extract 16]**: Well, I tried to stop a few things after that to increase my potency at large such us doing a lot of exercise, supplements e.g., some testosterone boosters. I even bought some Viagra online. But nothing actually worked (No. 4 225–227).

**[Extract 17]** Since about when I was 19, I have been having obsessive thoughts about my appearance, my height, how I feel I would have no chance with any girl I would be attracted to. In fact PMO^2^ was my escape from these feelings to that I could function as a normal human being for a little while after I ejaculated (No. 12, 1126–1129).

As it has been demonstrated so far, participants’ self-esteem is based on the internalization of both masculine and feminine idealized standards derived from hypersexualized culture, managed through pornography consumption, and enhanced by it. In these extracts, online users seem to draw on conceptualizations of shame as a strong motivator to “fix” themselves and to regain control over hegemonic masculinity.

The causal role of these factors could be understood within the social process of self- objectification within which the narrators’ account implies their attempt to restore their lost masculinity. The theory of self-objectification refers to the adoption of an observer’s gaze to evaluate one’s own body (Fredrickson, Roberts, Noll, Quinn & Twenge, 1998; Vandenbosch & Eggermont, 2013). Body surveillance, body dissatisfaction and body shame introduces participants into a vigilant monitoring of their outward appearance transforming their bodies into a project to work on. Participants resort to the adoption of appearance modification behaviors in order to increase their body-esteem aiming to a better sexual improvement in the long-term. These “stylized” set of acts stem from muscular standards, prevalent on mass media, and include regular body exercise and consumption of commercial commodities (e.g., Viagra, testosterone boosters) that aim to enhance sexual potency. However, this ongoing body surveillance seems to further result in sexual difficulties, largely contributing to the increase of pornography consumption. These master narratives confirm Taylor and Jackson's (2018) study on the involvement of masculinity as an internal and performed capacity in the expressed overwhelming lived experiences and Hartmann's (2021) hypothesis that connects the NoFap culture with the promotion of a sex-work ethic based on self-govern practices. It seems that the common rationale behind this attitude is the willingness to restore the male's biological capacity for heterosexual intercourse- participants' hybrid masculinity (Burke & Haltom, 2020).

Within this context, the participants’ fragile hegemonic masculinity adds to the complexity of causal connections between “being weak” in a hypersexualized culture and PPU. The portrayal of men as super-powered leaves them with an aggravated sense of inadequacy. Therefore, it could be argued that pornography consumption essentially promotes further indulging of participants into the seemingly safer, unreal world of pornographic fantasies.

Another possible etiological pattern showcased in the following extracts links PPU with narratives of an unfulfilling lifestyle. It could be argued, that male performativity was also extended from a sexually private space to a social, public area. In short, another dimension of the hegemonic masculinity involves participants’ ability to socially demonstrate and validate their masculinity:

**[Extract 18]**: I'm crying as of this writing, because it is such a pain. Now I've become almost useless and such a failure in life. Especially at that age, when the majority of my friends have good careers, some with their own businesses, most have girlfriends, and those who don't are at least not idiots like me (No. 10, 984–987) [Emphasis in original].

**[Extract 19]**: Currently, I have no release, just these thoughts of insecurity and helplessness in my mind, telling me no matter how much money I earn, how much time I spend in the gym, how good clothes I wear I will never have an opportunity with an attractive girl. I am easily triggered to this state when walking around in the city and noticing hot girls (always measuring up their boyfriends, comparing them to myself and feeling lacklustre) (No. 12, 1130–1134).

The aforementioned narratives offer a plethora of readings about the required skills needed in order for someone to be able to prove his masculinity both on a self- conscious level and socially. Participants’ narratives are framed by a “discourse of distress” which can be seen as the result of diminished self-worth and their self-conceptualization as socially inferior. Specifically, having a well-paid job, being well-dressed, fit, and in relationship with an “objectively” good-looking woman, corresponds to the socially recognized lifestyle symbols of happiness, desirability, and success. As a consequence, the absence of these social attributes render participants in vulnerable position of feeling inadequate and worthless. However, it is not merely the absence of these skills that affect men’s reputation but also their need for recognition of their personal value by other men.

The double meaning attached to the notion of male identity can be recognized within this emerging social interaction of male competitiveness. More specifically, participants seem to be both subject to a self-conscious identity and to others’ control and recognition. Thus, online users’ masculine identity is confirmed on a personal level by a successful sexual performance and socially by leading a specific lifestyle, which imposes finding a female partner. This practice could be interpreted in what Brooks (1995) called trophyism—demonstrating, both consciously and unconsciously, attractive women as sexual achievements in front of other men. Being promiscuous, results in the validation of the heterosexual identity and the enhancement of the status quo which, combined with the standards of credibility, leads a man to accomplish the standards of the idealized hegemonic masculinity. Hence, it could be argued that “others” gaze is deemed as more valuable than their own needs. As one participant noted: “If a person is completely without limits and succeeds in achieving everything they want, then the person becomes a perfect reflection of the world” (No. 25, 2247–2249).

Drawing on phenomenological theory of incorporation (Mason-Grant, 2004) in the context of being constantly related to others (people and groups), individuals’ bodies and attributes tend to become noticeable when a remarkable degree of similarity with others is achieved, in terms of acting and looking. In this way, a degree of normalcy and acceptance is structured. Within this context, feelings of shame, self-hatred and constant self-criticism emerged, followed by a prevalent narrative of self-perfectionism.

In the aftermath of unmet goals and failed expectations, pornography consumption came to be seen as the cause. The wasted hours of IP, hours that could have been spent “perfecting oneself.”

### “Storyfication Trap”

Within this part of the chronicle, a new narrative unfolds around a process of “gaining the superpowers back^3^.” Participants’ prolonged state of distress regarding such a core facet of oneself, like sexuality, is a powerful motivation for self-intervention. In this process, several behaviors emerged starting by the conceptualization of the “problem” that needs to be “addressed.”

**[Extract 20]**: My first actual attempt to sleep with a girl was two years ago. At this time I didn’t know that I am addicted to porn or knew about its horrifying effects it can cause. When I tried to have sex with her I was able to get a limpy errection during foreplay but not when I tried to stick it in. That was the moment where I almost freaked out because it was the first time I noted that something was wrong with me. After that I was afraid that I may have ED and when I read some things about it I also realized that I haven’t had a proper morning wood in month (maybe even years). Well but sadly I stumbled about porn addiction and that this may also cause ED after all (No. 4, 217–225).

From the above-mentioned narrative, it could be supported that online users’ ED is considered the crucial event that raises a series of concerns about pornography being problematic. This was also the starting point of a diagnostic process where the behavior of “comparing and fitting” into available online categories took place. In this context, addiction to pornography started becoming more of a self-identified problem. As Attwood (2006) puts forward, pornography addiction seems to be the only available explanation across the internet with regards to patterns of pornography consumption.

In that way, online pornography addiction communities emerged as the real “eye opener” by taking advantage of the position of “talking from lived experience.” Thus, the disease model within these communities was framed as a mutual concept providing a common understanding. However, in line with the view that individuals are active agents of discourse (Emerson & Frosch, 2004), it is believed that participants, by using a “discourse of addiction,” consciously place their resort to pornography within a “framework of justification.” More concretely, according to Thomas (2016), the addiction model is a symbolic resource that is usually evoked by individuals in order to mitigate feelings of guilt and responsibility.

As a consequence of blaming pornography for their distressing experiences, abstinence was promoted as the indispensable intervention. Within this context, abstinence was linked with the notions of recovery and relapse.

**[Extract 21]**: The difference between then and now is that now I know firsthand exactly what I have to lose. I know just how much beauty and opportunity and richness I will be throwing away if I do not remain committed to my recovery from porn. (…).The past 165 days of recovery have been, although markedly more challenging and painful than any other attempt in my life, equally as transformative. The problem is that transformation can quickly and easily dissolve into the abyss of suicidal despair if the porn addict pathways of the mind are revived (No. 29, 2698–2706).

**[Extract 22]**: I question the improvement in my recovery. I question my future. I worry repeatedly. Every day is tremendously stressful. I continuously have thoughts of porn. It's tough to use the PC without clenching my teeth and banishing a nasty urge (No. 30, 2726–2728).

**[Extract 23]**: And EVERY TIME, I relapse I get anxious and paranoid (for no logical reason, is it a guilt trip I'm stuck in?) (No. 23, 1858–1859) [Emphasis in original].

Participants’ stories of recovery and relapse are framed by dichotomous thinking. For instance, recovery has been associated with conceptualizations of “beauty, opportunity and richness” while relapse is experienced with overwhelming emotions and a sense of failure. When framed together, it could be argued that recovery symbolizes self-affirmation and faith to a brighter future, while failure to persevere reflects the possible loss of self. Moreover, since individuals are presented as unable to manage their choices within medically-oriented underpinnings, the commitment to recovery requires an ongoing focus on self-control (Thomas, 2016). Within this context, a new mentality emerges:

**[Extract 24]**: I am in my 400+ day streak on my first attempt after I choose to proceed into a lifestyle change. The fact that nofap is so rough challenged me. I wanted to succeed magnificently in a thing that so many people find so hard. It was very hard for me as well, but that made it easier. (…) I converted my PMO addiction to nofap addiction. That would be the toxically shame approach of superachiver: you can defeat an addiction by turning addicted to the pride of beating it (No. 25, 2112–2115, 2150–2152).

Specifically, this bodily intervention of abstinence was also followed by the transformation of the diagnostic identity; from a pornography addict to a nofapper. Abstinence has been regarded as a common goal reflecting a man’s reputation and value; almost monastic in some ways. But, having sexual outlets is integral to our species, meaning that abstinence for those not in sexual relationships is virtually impossible. It could be supported that nofap was transformed into a moral commitment that invests in the “price” of hegemonic masculinity. Within this context of promotion of performance ideals, abstinence was linked with an, “accreditation ceremony” (Bergner & Bridges, 2002) where competitiveness had formerly been a prevalent way of reassurance. In particular, the intense shame, inherent in narrations of sexual humiliation, is transformed into a “super-achiever approach” in homosocial environments of mutual understanding. Here, the “triumph” among male nofappers is the confession of “success stories” which is linked with repeated and compulsive engagement into recovery streaks. In contrast, partial “triumph” or relapse lead to constructs of failure and worthlessness not only on a personal level but also as a member of a group. Taking into account the interpersonal level that participants’ storytelling is situated and formed, the dialectical relationship between narrators and the audience reproduces and relies on a public narrative that links abstinence with time management and self-control. This material conditioning incorporates and refers to socially valued discourses of “success” and “worth” producing the participants’ ontological narratives of discipline and hard work that are embedded in the concept of addiction. These conceptualizations provide more thorough understandings of the narrators’ meaning-making process in their self-perceptions of PPU. Thus, over-emphasizing on the notions of recovery and relapse seems to exacerbate feelings of despair and therefore further perpetuates PPU.

**[Extract 25]:** By believing in the story I shaped around and about addiction to porn, I sustain this energy, and use more energy trying to fight my own creation i.e. I keep the thought cycle going in my mind about how I am an addict, how long I been doing it, how far I went, how life sucks because of what I did, that I must face withdrawal and a hard battle ahead. When I realized this storyfication trap I was keeping my attention in, I was no longer in and part of the story, but was outside of it, looking in…This brought a great sense of relief, I am not my story! What story are you telling yourselves? Look it over, and see who are you without this story? (No. 18, 1461–1470).

**[Extract 26]:** Reading all of those success stories and expecting enormous changes in my life merely because I was quitting porn transformed into a kind of mental masturbation for me. I put too much pressure on myself to reach this goal—and the goal was superficial. I wanted “superpowers,” and I believed that Nofap was this particular thing keeping me from happiness. Now, I have a healthier approach (No. 7, 610–614).

What is observed for some participants is that there is a “narrative of change.” That is, how the participants’ sense-making process and self- conceptualizations could be transformed as well as how these changes are framed. Thus, at this point, a narrative re-construction emerges (Jirek, 2017). Specifically, participants’ narrative of change came along with the realization that this framework of pornography addiction fails to provide adequate insight for their distress. In particular, its reduction of the lived experience to biophysical parameters fails to provide an adequate explanation of the changing relationship between themselves and the social world.

By questioning “who are you without this story?” participants essentially challenged the discourse provided on the forums and, by extension, their self-diagnostic labels. Additionally, this narrative re-construction portrays their effort to frame the etiology of their PPU within a complex socio-psychological interaction. Specifically, narrators locate the source of their PPU in a conflict between their personal identity, social norms, and expectations from life.

## Discussion

The analysis of narratives of online r/Nofap and r/PornFree reddit users with self-reported PPU revealed multiple etiological connections, informed by a range of intrapersonal, interpersonal and socio-cultural narratives related to the onset of PPU. At first, the interaction between materiality, the embodied spectator, and a perceived hypersexualized culture contributed to the creation of an “online persona,” signifying the beginning of the PPU experience. Furthermore, the narratives of an adverse childhood, inadequate gender performativity, and an unfulfilled lifestyle formed a belief of “being weak” resulting in distress. The causal contradiction between a psychological state of “being weak” and an empowered online persona portrayed the IPU as a means of escape and validation. Finally, dichotomous thinking, linked with the notions of recovery and relapse, rendered abstinence as a valuable intervention in a context of powerlessness.

The narrative analysis revealed the crucial contribution of adverse life events to the onset of PPU, highlighting dissociation through pornography as a defense mechanism; partly keeping with the findings of Ybarra and Mitchel (2005), who suggested childhood trauma as a basic causal link to the development of PPU, and Cavaglion (2009), who suggested a history of isolation and solitude. In this study, participants’ intentional IPU was framed by motivations of escape, security, control, sexual and social success. Therefore, it can be supported that frequent pornography consumption does not seem to act like some typical dissociative facilitators (like alcohol or drugs). In particular, instead of numbing painful emotions through the facilitation of self-detachment, pornography offers the potential for transforming the self into a desired subjectivity within a virtual reality. In short, individuals have the opportunity to escape from a painful reality and resort to an artificial one where their wishes for transformation into ideal multilevel subjectivities are fulfilled through fantasy.

In addition, previous literature has attempted to showcase the mediating role of religiousness (Grubbs et al., 2017) in constructions of pornography addiction. Although these factors have been an important counter-argument against the medicalization of this behavior, their etiological influence on participants’ self-perceptions of PPU seems limited. Unlike these interpretations, this study offered insights regarding the crucial role of unsuccessful sexual performance as a potential causal pattern in the onset of self-perceived pornography addiction. This differentiation is likely to be a result of the fact that the sample itself seems less religious than the general population. In a recent survey, the founder of both Nofap and r/Nofap, Rhodes (2018), highlighted the non-religious character of the NoFap community, presenting that 62% of online users declare themselves as non-religious. In this context, religious discourses, such as demonizing the situation, were used alongside notions of addiction. Together, they formed a useful framework of justification that enabled them to maintain some sense of integrity and autonomy in a context of powerlessness. Therefore, it could be supported that conceptual constructions such as gender performativity, ongoing self-improvement, and successful social exhibitionism, when attached to an identity crisis, appeared to play a vital role in self-perceptions of pornography addiction.

Moreover, it has been observed that moral incongruence emerged as an etiological pathway of distress (Grubbs et al., 2019), consistent with the analytical outcomes of this study, when it was linked with the notions of recovery and relapse. In asserting this position, as these notions were framed by a dichotomous thinking of failure and prosperity, abstinence emerged as a collective moral commitment (Buckingham, Frings, & Albery, 2013). Here, the qualitative differentiation between moral incongruence and abstinence as a “collective moral commitment” needs to be highlighted. The discourse of distress after IPU, does not appear as a consequence of moral deviation that draws upon religious discourses and provokes intense feelings of guilt and shame. Instead, participants’ accounts of distress are associated with a rigid culture, promoted by the r/Nofap, within which procedures of abstinence are linked to claiming of male honor and its violation with intense shame, accompanied by feelings of betrayal and inferiority. Drawing on Dingle, Cruwys, and Frings’ (2015) work on social identity and addiction frameworks, the feelings of unmet identity needs when combined with social isolation, reinforce motivations for improving a fragile self-image. Consequently, in the context of promoting mutual support and self-control, the transition from a pornography addict to a no-fapper identity contributes to a sense of belonging. The emerging competitiveness among members provides a sense of power over the others, further enhancing feelings of achievement. Hence, the “acquired” nofapper identity (Dingle et al., 2015) implies a mutual moral commitment to “regain” hegemonic masculinity and well-being. Thus, any relapse is in stark contrast with participants’ sacred values and all the more devastating.

### Implications for Clinical Practice

This study provides valuable insights that could be employed in a clinical and therapeutic domain. The causal complexity between social norms, interpersonal interactions, personal motivations and expectations underlines the need for clinicians to evaluate the etiological variants of PPU beyond biomedical conceptualizations. Specifically, the analysis indicated that the narratives of distress around PPU are possible manifestations of internal conflicts. Since these conflicts stem from a causal interaction between transformations of the self, identity loss, social expectations, and an adverse interpersonal history, clinicians may need to examine the motivations, personal meanings and functions attached to the use of IP. In this study, for instance, the participants’ concerns regarding sexual performance are portrayed as vital for the onset of their self-perceived PPU. This finding questions the current biomedical model where sexual difficulties are not deemed as a main factor contributing to excessive pornography consumption, rather than a symptom of pornography addiction (Tylka, 2015). Therefore, clinicians could assess presented complaints of diminished well-being (depression, anxiety, socialization) in relation to internalized gender ideals.

Additionally, therapeutic approaches to PPU that usually draw on interventions for sexual addiction, typically involve self-regulation by identifying the environmental triggers that enhance sexual urge, and/or techniques which facilitate self-observation (Goodman, 1993; Rosen & Leiblum, 1995). However, since the perceived gender invalidation was followed by a set of self-objectification practices (e.g., body surveillance and bodily interventions), which is linked both to meta-performance anxiety (especially for individuals with reported sexual performance difficulties) and to abstinence procedures (which applies to both subgroups), it could be argued that a therapeutic intervention emphasizing on these aspects would further fuel anxiety and the need for self-improvement.

The present study, in general, contends that pornography use could, indeed, become problematic with multiple psychological implications. However, PPU seems to arise and be maintained by vicious cycles of causal connections. In particular, the finding highlighting a utilitarian use of pornography demonstrates the different forms of individual use. For instance, motivations of escape and/or validation might indicate one’s overall anxiety in relating with others. Thus, IP may acts as a means for wishes to be fulfilled and experienced as a reality, suggesting a need for affirmation and interaction with others that takes place in a less threatening and more controllable virtual environment (Thomas, 2016). These potential causes might also provide an understanding of those experiencing profound distress and having difficulties or willingness to cease this behavior. In clinical settings, being able to differentiate between these etiological variants would potentially facilitate a more targeted and individualized intervention. Thus, a thorough investigation of the development of PPU is considered as highly important as are identifying maintenance feedback loops. On this basis, interventions aimed at managing the frequency of pornography use are considered as less important.

Concerning treatment modalities, it could be argued that a narrative psychotherapeutic approach could be effective, as it focuses on providing an understanding of the psychological process of meaning-making and its role in individuals’ lives (McTighe, 2018). Therefore, it is claimed that a joint approach in “re-writing” the narrative history would enhance individual’s feelings of being accepted and understood. Narrative therapy was traditionally utilized in working with identity (Plummer, 1995) and relational difficulties (Elbert, Schauer, & Neuner, 2015; Maarof, Hashim, Yusof, & Mydin, 2012). Thus, as intrapsychic factors relating to identity and interpersonal difficulties (relational problems, neglect, mistrust) were involved in the onset of PPU, narrative therapy is considered an appropriate therapeutic approach. Alternatively traditional cognitive behavioral approaches could be utilized. As can be seen in Fig. 1 there are clear indications of the role of cognition in both developing and maintaining PPU. Focusing on the content of cognition regarding both ideas of masculinity and being weak may well lead to a change in behavior. In addition, if IP is indeed involved in the management of distress then behavioral interventions based upon healthier options could be considered. Third wave cognitive behavioral therapies, such as acceptance and commitment therapy (Crosby & Twohig, 2016), may be useful with helping with the crisis of identity through confirming values and working towards acceptance and behavior change.

### Limitations

The present qualitative study is also subject to particular limitations. First and foremost, the collection of the data through online forums limited the researcher’s capacity for seeking further clarification on lived experiences (Holtz, Kronberger, & Wagner, 2012). Thus, the examination of the causality of PPU through interviewing participants would give valuable insights allowing for a more thorough understanding of events and experiences.

Additionally, a relatively homogenous sample is evident from the available demographics derived from the participants’ personal narrations. In particular, the fact that the research participants were heterosexual, mostly single men limits the generalizability of the present findings to more diverse populations. Therefore, future research could explore the etiology of PPU in both women and non-heterosexual populations, since social identity status is considered as a crucial factor in how distress is expressed and experienced (Tylka, 2015). Moreover, the particularly young age of participants (19–30) limits the extrapolation of the findings to the whole male population. It is possible that a wider spectrum of age would have revealed significant differences in individual's motivations for engagement in IP. Additionally, it is believed that access to more elaborate demographics such as social class and ethno-cultural background would contribute to a better understanding of individuals’ voices regarding the role of social context in the etiology of PPU.

Finally, another limitation in terms of the representativeness of the study’s population is linked with the normative structured self-help communities advocating pornography abstinence. Since the role of addiction is prevalent in the narratives of r/Nofap and r/Pornfree, it could be argued that participants’ choice of events and the way of its narration are largely influenced by these conceptualizations. Hence, the participants of this project could be considered a niche population, highlighting the necessity to investigate the etiology of PPU by exploring narratives collected from non-addiction oriented environments.

### Conclusion

This study aimed to contribute to the scholarship regarding PPU emphasizing the voices of IP users and moving beyond discourses of “harmful effects.” To our knowledge, this study is one of the first attempts to directly explore the etiological variants of self-perceived PPU of the NoFap/Pornfree population using a detailed qualitative analysis of their lived experiences.

The narrative analysis revealed that conceptualizations regarding the etiology go beyond reductionist interpretations of “cause and effect.” The causal efficacy between intrapersonal, interpersonal and contextual aspects forms a complex network of etiological interactions. The participants’ narratives suggest that the onset of PPU was determined from a constant self-negotiation between embodied relationalities and intersectionalities. In particular, the utilitarian use of pornography portrayed a background of relational marginalization and invalidation where intense feelings of a vulnerable real-self emerged. Similarly, individual’s inability to respond to a perceived hypersexualized culture, where gender expectations, self-actualization, and performance have replaced living itself, contributed to self-stigmatization and identity loss. Thus, in a context where the dialectical relationship between materiality and an online persona allows for free sexual expression only in a private space, IPU, apart from temporarily numbing negative feelings, serves, also, as a guise of coherence to a weak and fragile identity. On this basis, abstinence in online communities was connected with hope for a better future self. In this case, hoping implies a desire to regain the “lost hegemonic masculinity.”

Finally, this study highlighted the need both for a comprehensive assessment of the etiological spectrum of PPU in clinical settings and for further investigation in a more general population, moving beyond a conceptualization of addiction by incorporating self-perceptions of PPU into the individuals’ meaning-making processes.

## Footnotes

1 The word “fap” is an onomatopoeia used as a synonym for masturbation.

2 Pornography, Masturbation, Orgasm.

3 Discredited theories of semen retention leading to heightened testosterone levels (Pham et al., 2016).
